# Supplementary figures and images for: Excitatory to inhibitory synaptic ratios are unchanged at presymptomatic stages in multiple models of ALS
Source: PLoS One. 2024 Aug 1;19(8):e0306423. doi: 10.1371/journal.pone.0306423 (PMC11293752; doi:10.1371/journal.pone.0306423)

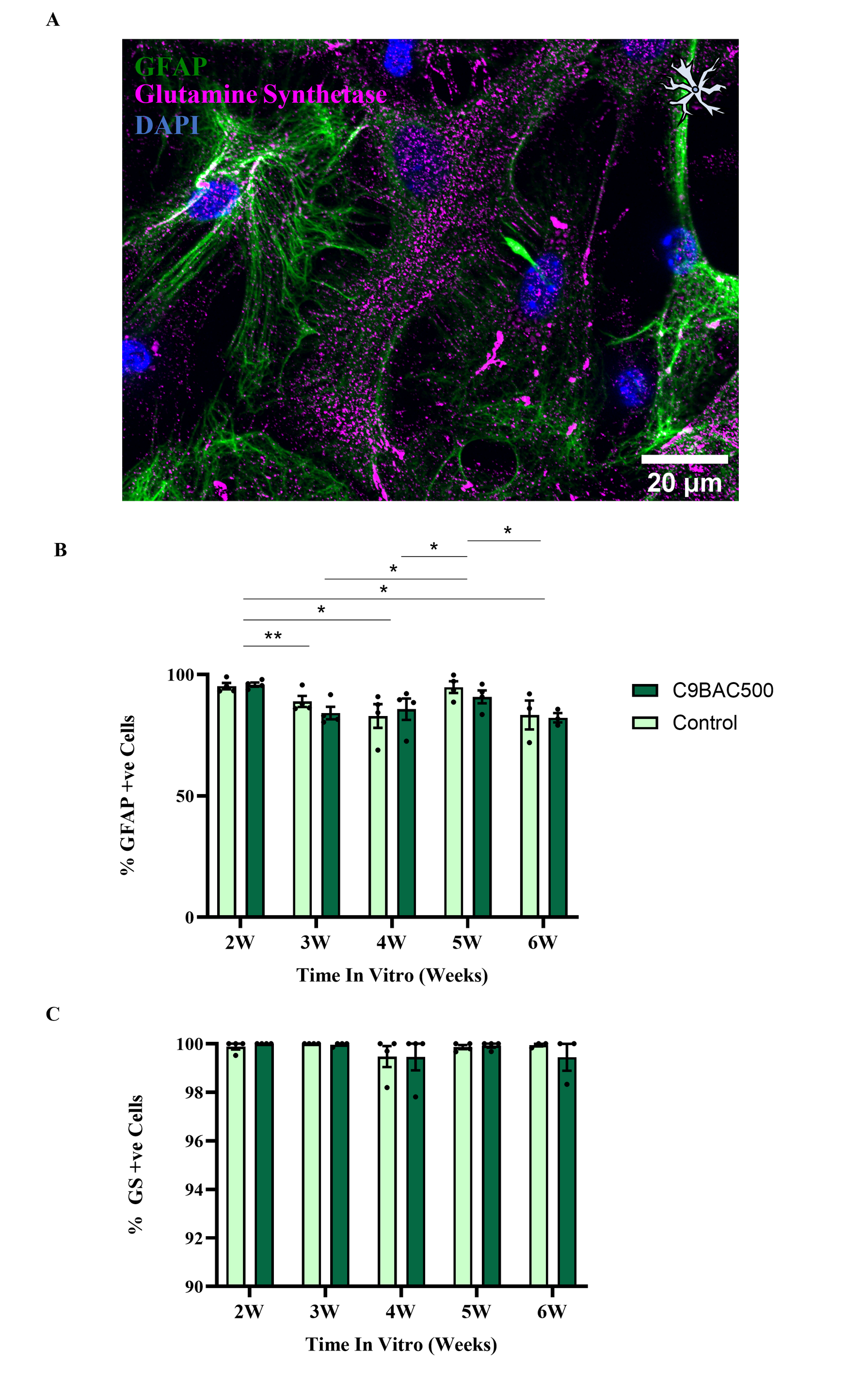

Supplement: S1 Fig — A) Representative image demonstrating GFAP and glutamine synthetase (GS) in enriched astrocyte cultures. B) Quantification of % cells positive for astrocyte marker GFAP at 2–6 weeks in vitro. C) Quantification of % cells positive for astrocyte marker GS at 2–6 weeks in vitro. (TIF) [file pone.0306423.s001.tif]

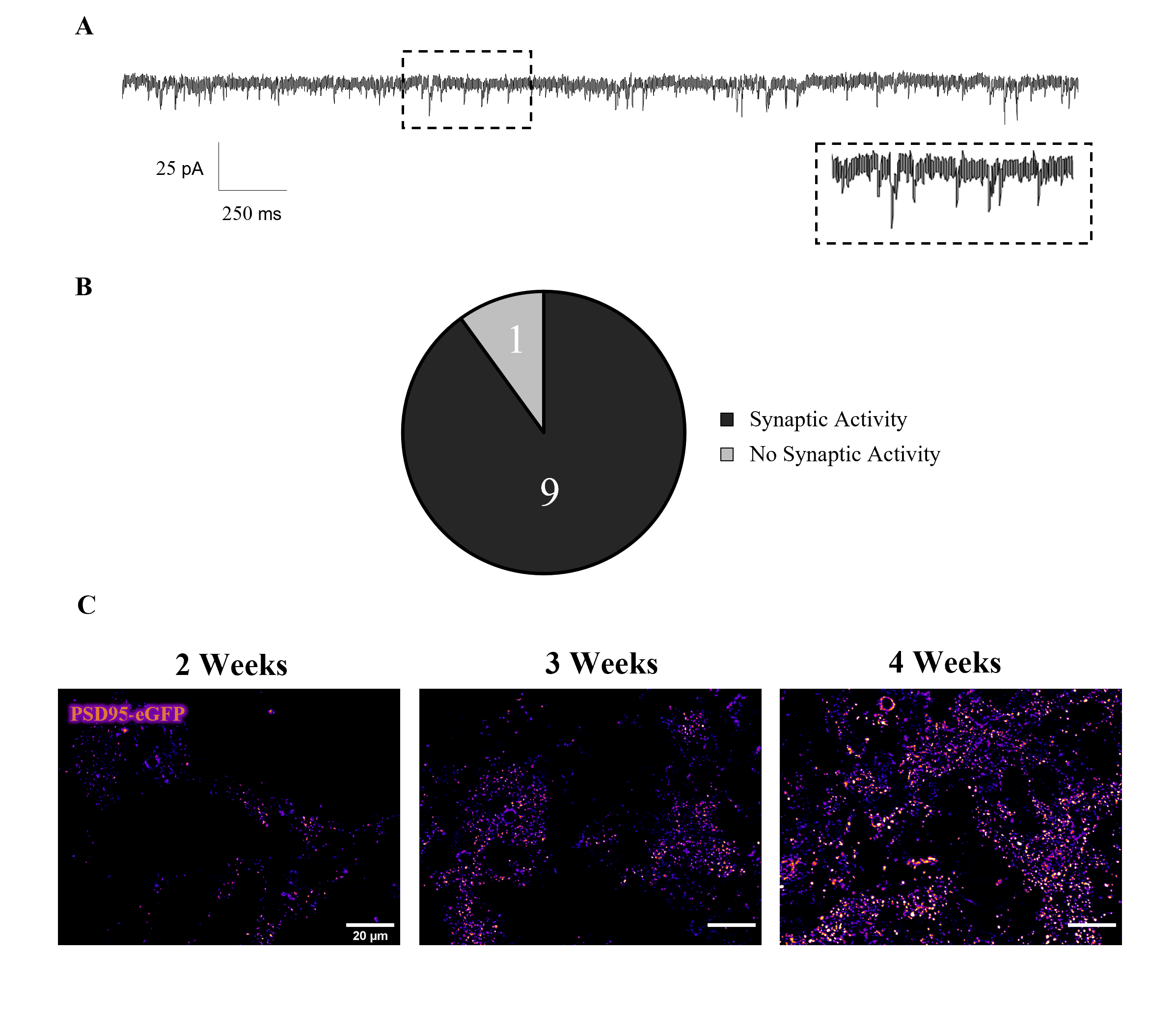

Supplement: S2 Fig — A) Example whole-cell patch-clamp recording demonstrating spontaneous synaptic activity measured in voltage-clamp mode in spinal neuron cultures at DIV 18. B) Pie chart demonstrating the proportion of cells receiving any spontaneous synaptic input versus no clear synaptic input in a 120s gap-free recording in voltage-clamp mode. C) Examples of PSD95 expression at 2, 3 and 4 weeks in vitro. (TIF) [file pone.0306423.s002.tif]

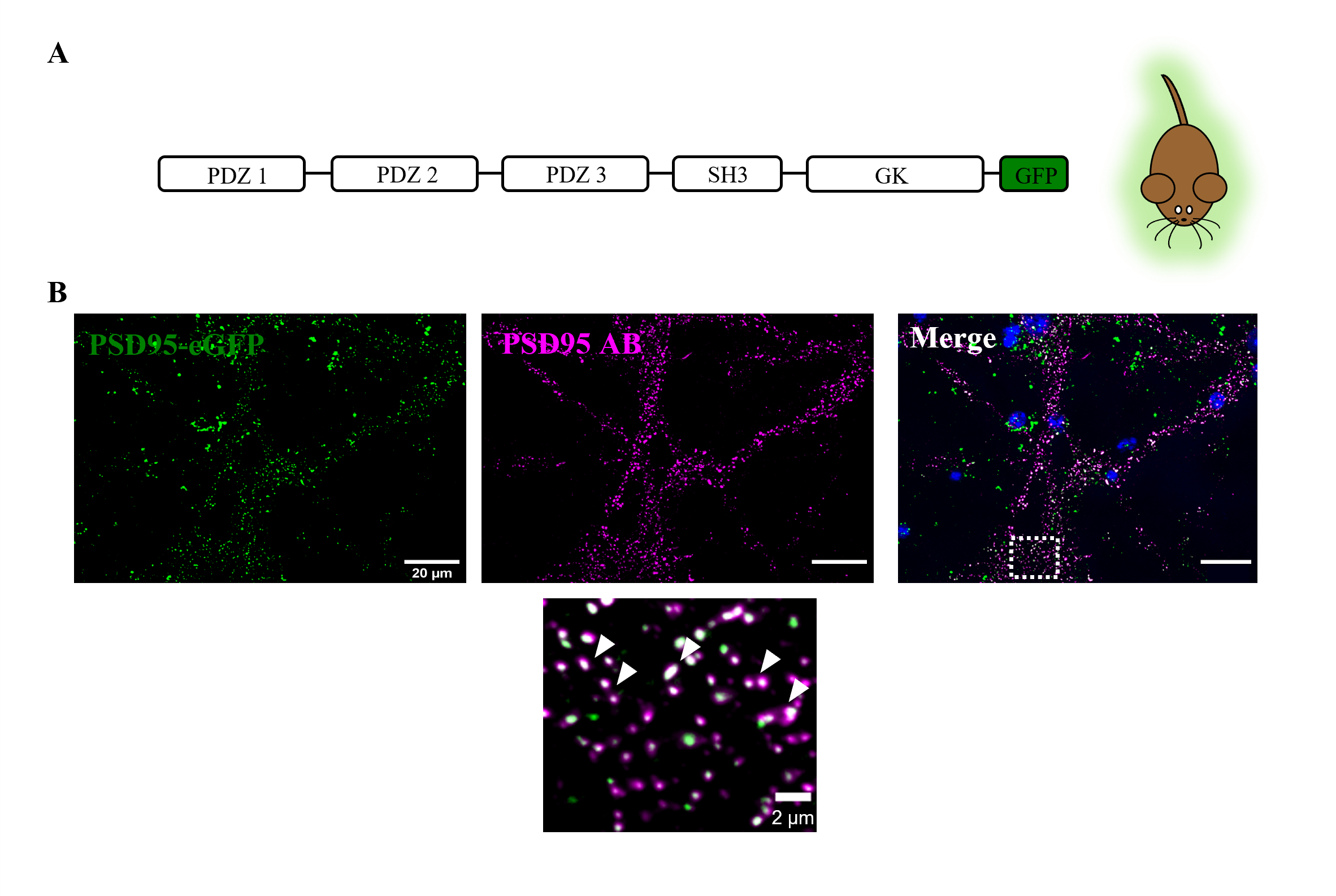

Supplement: S3 Fig — A) Schematic demonstrating the structure of PSD95-eGFP. B) Validation images in C9BAC500+/- PSD95-eGFP+/- neuron cultures stained with an anti-PSD95 antibody (PSD95 AB). White arrows indicate clear co-localisation between GFP and PSD95 AB. (TIF) [file pone.0306423.s003.tif]

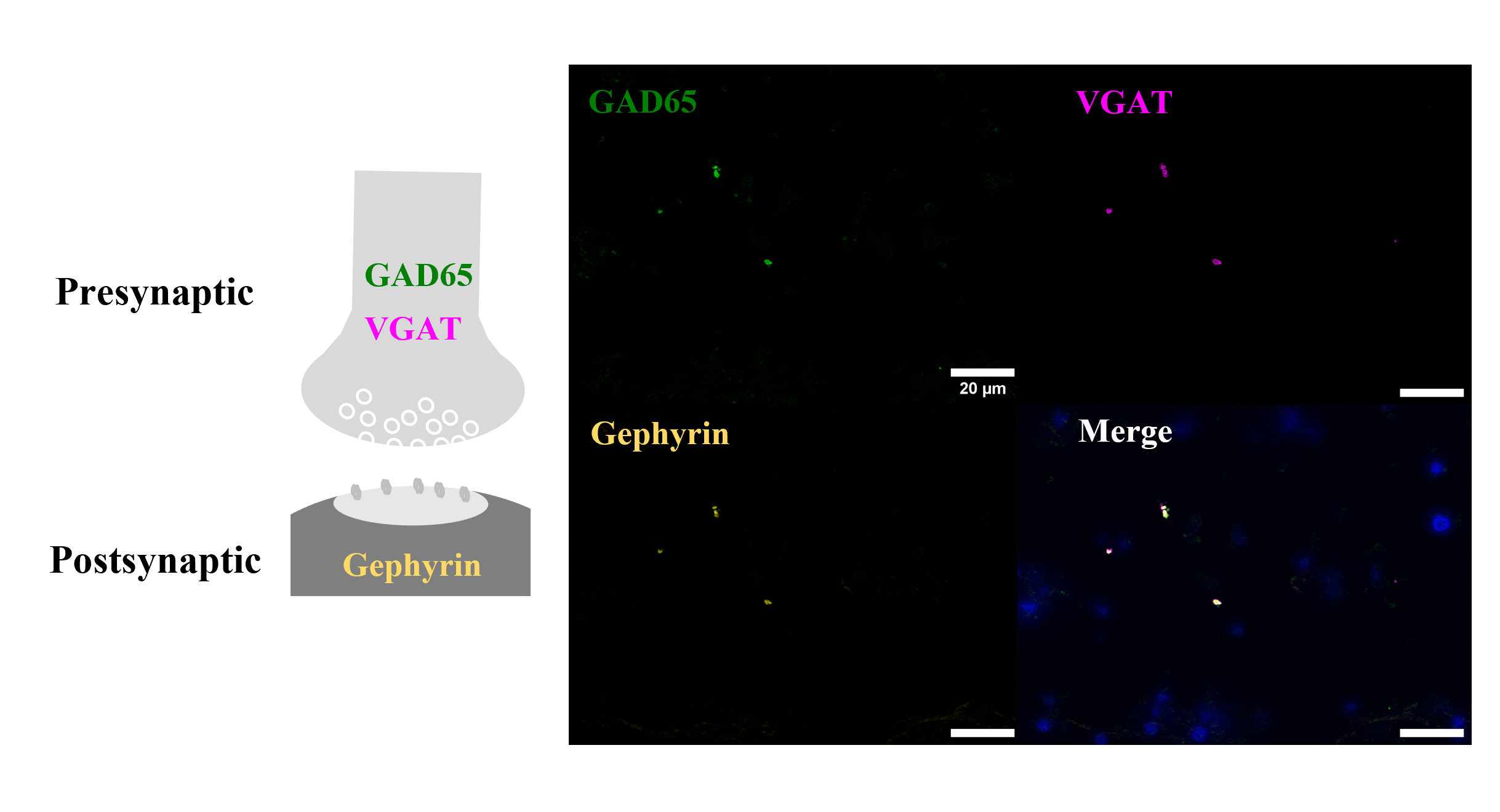

Supplement: S4 Fig — Schematic demonstrating expected location of GAD65, VGAT and gephyrin expression, accompanying an example image of co-localised presynaptic inhibitory markers GAD65 and VGAT, overlapping with postsynaptic marker gephyrin. (TIF) [file pone.0306423.s004.tif]
