# Supplementary material for: Excitatory to inhibitory synaptic ratios are unchanged at presymptomatic stages in multiple models of ALS
Source: PLoS One. 2024 Aug 1;19(8):e0306423. doi: 10.1371/journal.pone.0306423 (PMC11293752; doi:10.1371/journal.pone.0306423)
Supplement: Attachment — Submitted filename: Response to Reviewers .docx [file pone.0306423.s005.docx]

Response to Reviewer

We would like to thank both reviewers for their careful consideration of the manuscript, and for acknowledging the importance of our findings for the field. We have now amended the manuscript in light of the helpful reviewer comments. We believe the changes made have improved the manuscript and hope it will satisfy the requirements for publication. In our responses below, and when referring to lines or sections, we refer to changes in the ‘tracked changes’ version of the manuscript.

Reviewer 1:

*“The only thing that I would like to see would be the inclusion of some details of the phenotypes of the mice that they are using. Whilst I am aware most of these are published models, it would be better to have the details in this paper so that people can see how early these are being studied before symptoms*.”

- We agree that providing a more thorough background on the phenotypes of the mouse models is highly valuable and aids in the interpretation and understanding of our findings. We have therefore provided more substantial information regarding the phenotypes of the mice in our revised Discussion section [431-444].

“Again for the iPSC, give some phenotype details so that it is clear what is being actually looked at here in terms of time frames etc. Given these are validated models, do these iPSC show phenotypes that should be taken into account.”

- Similarly, we have provided more extensive discussion of the phenotypes of iPSC-derived MNs [509-515]. Notably we have included mention of a hyperexcitability phenotype we (and others) have previously seen in iPSC-derived MNs at the timepoint used in our current study. This provides further support for our conclusion that any observed hyperexcitable phenotype is unlikely to be driven by biases in excitatory versus inhibitory synapse formation [396].

Reviewer 2:

*“It seems surprising that the E:I ratio is shown first, followed by the counts of the different synapses. It is expected that the synapses have already been counted, in order to get the ratio..? Why not show these data first, and then the ratio as the last part? In fact, it should be clarified in details how exactly the ratio was calculated, as that is not clear in the present version.”*

- We quantified the number of excitatory and inhibitory synapses in each individual image, then an E:I value was produced from these values (no. excitatory synapses / no. inhibitory synapses). The ratios for each image were then averaged across mouse or cell line. A more detailed explanation of E:I quantification has been added in the revised Results section [287-289].
- We appreciate that these data could be presented in either order (i.e. raw synapse numbers followed by ratios or vice versa). We wanted to present the ratio data first because we think it provides the clearest and simplest summary of the data, and speaks most directly to the main question we set in our project; whether the balance between excitatory and inhibitory synapses is altered at early stages of disease. Subsequently, we show the number of excitatory and inhibitory synapses (normalised to either the area or the number of DAPI nuclei) to verify that the static E:I ratios observed were not masking a scaled increase / decrease of excitatory and inhibitory synapses. We have added a justification for why we present the ratios followed by synapse densities in our revised Results section [285-287].

*“Based on this explanation, it would be most suitable to display the data derived from -N/-A conditions first in the figures, since that would be close to normal situation, with no mutations expressed. Usually, when preparing figures, the wild-type or control situation is displayed first, followed by the different interventions of increasing complexity. This comment is relevant for all figures.”*

- All relevant figures (Fig. 1, 2, 3, 4, 5 and Supp. Fig. 1) have been amended accordingly.

*“Figure legends could be improved by adding a short sentence in the beginning, as sort of a header. This would help the reader get the conclusion from each figure.”*

- All figure and supplementary figure legends now include an initial title sentence.

*“Page 5, line 123. Please specify the specific “C57bl/6” background, as there is more than one strain of these mice.”*

- We have now included full details of the mouse lines [124-128].

*“Page 13 line 299 refers to “Figure 1D”, but there is no panel 1D. Similar in the Figure legend above on page 12. Again, this comment is also relevant for Figure 2. Please correct.”*

- We thank the reviewer for noticing this error. Panel denotation for Figure 1D and 2D have been added to the figures.
